# Heyde Syndrome Unveiled: A Case Report with Current Literature Review and Molecular Insights

**DOI:** 10.3390/ijms252011041

**Published:** 2024-10-14

**Authors:** Mladen Maksić, Irfan Corović, Isidora Stanisavljević, Dušan Radojević, Tijana Veljković, Željko Todorović, Marina Jovanović, Nataša Zdravković, Bojan Stojanović, Bojana Simović Marković, Ivan Jovanović

**Affiliations:** 1 Department of Internal Medicine, Faculty of Medical Sciences, University of Kragujevac, Svetozara Markovica 69, 34000 Kragujevac, Serbia; asussonicmaster95@gmail.com (M.M.); radojevicdusan@yahoo.com (D.R.); todorovic_zeljko@hotmail.com (Ž.T.); marinna034@gmail.com (M.J.); natasasilvester@gmail.com (N.Z.); 2 Center for Molecular Medicine and Stem Cell Research, Faculty of Medical Sciences, University of Kragujevac, Svetozara Markovica 69, 34000 Kragujevac, Serbia; ira.corovic@gmail.com (I.C.); isidorastanisavljevic97@gmail.com (I.S.); ivanjovanovic77@gmail.com (I.J.); 3 Department of Pediatrics, Faculty of Medical Sciences, University of Kragujevac, Svetozara Markovica 69, 34000 Kragujevac, Serbia; tijanaveljkovic96@gmail.com; 4 Department of Surgery, Faculty of Medical Sciences, University of Kragujevac, Svetozara Markovica 69, 34000 Kragujevac, Serbia; bojan.stojanovic01@gmail.com

**Keywords:** Heyde syndrome, aortic stenosis, angiodysplasia, von Willebrand factor, acquired von Willebrand deficiency

## Abstract

Heyde syndrome, marked by aortic stenosis, gastrointestinal bleeding from angiodysplasia, and acquired von Willebrand syndrome, is often underreported. Shear stress from a narrowed aortic valve degrades von Willebrand factor multimers, leading to angiodysplasia formation and von Willebrand factor deficiency. This case report aims to raise clinician awareness of Heyde syndrome, its complexity, and the need for a multidisciplinary approach. We present a 75-year-old man with aortic stenosis, gastrointestinal bleeding from angiodysplasia, and acquired von Willebrand syndrome type 2A. The patient was successfully treated with argon plasma coagulation and blood transfusions. He declined further treatment for aortic stenosis but was in good overall health with improved laboratory results during follow-up. Additionally, we provide a comprehensive review of the molecular mechanisms involved in the development of this syndrome, discuss current diagnostic and treatment approaches, and offer future perspectives for further research on this topic.

## 1. Introduction

**Figure 1:**
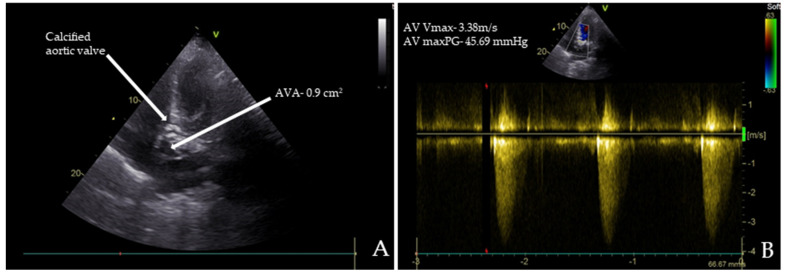
Transthoracic echocardiography (TTE) images of the patient: (**A**) TTE reveals severe aortic stenosis with a calcified aortic valve (AV) and an aortic valve area (AVA) of 0.90 cm²; (**B**) the maximum peak gradients (AV maxPG) across the AV were 45.69 mmHg, and the maximum systolic flow velocities (AV Vmax) were 3.38 m/s.

In the mid-20th century, Edward C. Heyde identified a link between gastrointestinal bleeding and aortic stenosis in patients over the age of 65, which he published in 1958 in the New England Journal of Medicine [1]. Subsequent research confirmed the presence of intestinal angiodysplasia, primarily in the ascending colon and cecum, which led to bleeding. Additionally, these patients exhibited an acquired von Willebrand factor deficiency (AVWD) type 2A, resulting in a hemostasis disorder and anemia [2]. A five-year study by Michael J. Krier and Omar Alshuwaykh further established the correlation between aortic stenosis and gastrointestinal bleeding in patients with intestinal angiodysplasia [3]. Heyde syndrome, with a prevalence of about 1% in the American population, is a very rare condition. The frequency of patients with both angiodysplasia and aortic stenosis ranges from 2–10%, with the true incidence believed to be much higher [4]. It has been shown that nearly 80% of patients with aortic stenosis have AVWD type 2A [5]. AVWD plays a critical role in the development of hemorrhagic diathesis. Its onset is explained by the passage of von Willebrand factor (VWF) through stenotic aortic valves, causing conformational changes in the VWF multimer. This exposes the A2 domain to the enzyme *ADAMTS13*, which breaks down VWF into smaller fragments, disrupting angiogenesis inhibition and the distribution of coagulation factor VIII (Figure 1) [6]. 

The diagnostic approach for Heyde syndrome is multidisciplinary, involving gastroenterologists, hematologists, cardiologists, and cardiac surgeons. In addition to laboratory analyses, it is necessary to perform echocardiography, endoscopic examinations, angiography of abdominal blood vessels, and video capsule endoscopy if previous diagnostics are inconclusive [7,8,9]. Although no official guidelines exist, the therapeutic approach includes blood transfusion, endoscopic therapeutic procedures, and transcatheter aortic valve replacement (TAVR) as the first therapeutic option [10,11].

The poorly understood connection between the conditions that constitute Heyde syndrome, combined with non-specific symptoms, can lead physicians to treat each condition separately, resulting in delayed diagnosis. Furthermore, the limited sensitivity of endoscopy in detecting angiodysplasia, along with the frequent absence of abnormalities in routine coagulation tests, can obscure the clinical picture and complicate the diagnosis [7,8,9]. Additionally, the unavailability of invasive treatments, patient refusal, or medical contraindications present further challenges in managing this syndrome. This underscores the importance of a multidisciplinary and individualized approach to patient care.

Therefore, we present a case of Heyde syndrome characterized by gastrointestinal bleeding, which was effectively diagnosed and managed through a multidisciplinary approach using endoscopic hemostatic procedures, blood transfusions, and iron supplementation. Additionally, we conducted a comprehensive literature review on the pathogenesis, clinical presentation, diagnosis, and treatment of Heyde syndrome, with a special emphasis on the underlying molecular mechanisms.

## 2. Case Presentation

A 75-year-old man presented to the emergency department at the University Clinical Center of Kragujevac with symptoms of malaise, exertional dyspnea, and melena. These symptoms began three months earlier and gradually worsened, with a significant deterioration in the past week and the onset of melena occurring one day before hospitalization. During this three-month period, the patient reported a steady decline in functional status, experiencing increasing difficulty in performing daily activities such as walking and climbing stairs, due to worsening fatigue and shortness of breath. Consequently, his quality of life deteriorated, marked by reduced mobility, more frequent rest breaks, and a diminished ability to participate in social and recreational activities. His medical history included type 2 diabetes mellitus, managed with oral antidiabetic medications, and untreated anemia of unknown origin. He had no history of drug allergies, tobacco or electronic cigarette use, alcohol consumption, or illicit drug use. 

Upon admission (Day 0), his vital signs were stable: heart rate, blood pressure, respiratory rate, and body temperature were within normal limits. Physical examination revealed pale skin and conjunctiva, mild bilateral pretibial pitting edema, a systolic murmur, and dark stool during rectal examination. The remainder of the physical examination was unremarkable. The laboratory workup demonstrated severe iron deficiency anemia, indicated by a hemoglobin level of 60 g/L, along with elevated concentrations of N-terminal pro-brain natriuretic peptide (NT-proBNP) (Table 1). Coagulation studies, including prothrombin time (PT), partial thromboplastin time (PTT), and fibrinogen, were within normal limits. Renal and liver function tests, electrolytes, tumor markers, and C-reactive protein were all within reference ranges.

Chest radiography, abdominal ultrasound, and upper endoscopy showed unremarkable results. Echocardiography revealed paradoxical low-flow, low-gradient severe aortic stenosis (Figure 1A,B).

**Figure 2:**
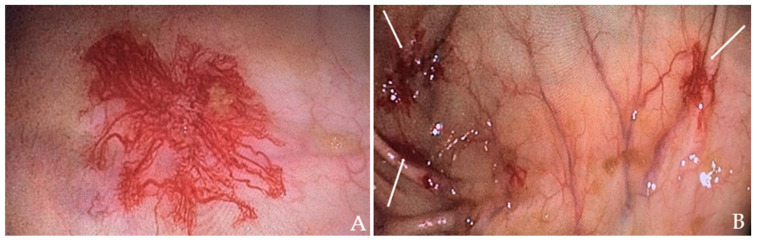
Colonoscopy images of the patient: (**A**) Angiodysplasia in the ascending colon. (**B**) Angiodysplasia in the cecum (indicated by the white arrows).

The patient received a transfusion of two units of concentrated RBC and low-dose diuretic therapy. Within two days, his symptoms improved, including a reduction in leg edema. A subsequent colonoscopy revealed angiodysplasias in the right colon (Figure 2A) and cecum (Figure 2B), which were effectively treated with argon plasma coagulation (APC).

Following a hematologist consultation, we performed testing for VWF antigen, VWF ristocetin cofactor activity (VWF:Rco), factor VIII, and the VWF antigen/VWF:Rco ratio. The results showed a decreased VWF:Rco/VWF antigen ratio (Table 1) with normal values for VWF antigen, VWF:Rco, and factor VIII levels, indicating AVWD type 2A. After being diagnosed with Heyde syndrome, the patient was referred to a cardiac surgery team that recommended a TAVR procedure; however, the patient declined the recommendation. During the remainder of his hospitalization, the patient received intravenous iron and showed gradual improvement in his symptoms, with no further episodes of melena. He was discharged on Day 5 with a hemoglobin level of 86 g/L (Table 1) and was prescribed oral iron and vitamin C supplementation, along with cardiologist-recommended therapy (angiotensin-converting enzyme inhibitor, beta-blockers, diuretics).

At a follow-up appointment two months later, his hemoglobin level had increased to 112 g/L, and NT-proBNP levels had decreased to 679 pg/mL (Table 1), with no recurrence of melena and significant symptom improvement. His functional status had notably improved, allowing him to return to near-normal activity levels and perform daily tasks with minimal exertion. The patient also reported reduced fatigue, increased mobility, and an overall sense of well-being. Given the patient’s clinical improvement and favorable changes in laboratory parameters related to anemia, a multidisciplinary team consisting of a cardiologist, gastroenterologist, and hematologist selected a “watch and wait” approach, continuing with conservative management. The primary focus is on correcting the anemia and preserving optimal cardiac function. The patient’s clinical status and laboratory parameters will be closely monitored through bimonthly follow-ups, with periodic echocardiographic and endoscopic evaluations integrated into the comprehensive care plan.

## 3. Discussion

### 3.1. Molecular Insights into Heyde Syndrome: The Role of VWF

#### 3.1.1. Structure and Function of VWF

VWF is a multimeric glycoprotein involved in hemostasis, functioning as a mediator for platelet adhesion and a carrier for coagulation factor VIII [12]. Synthesized in endothelial cells and megakaryocytes, the 220 kDa VWF monomer assembles into large multimers, with sizes reaching up to 20,000 kDa. Key interactions include the N-terminal D’D3 domains, which facilitate binding to the platelet GPIb receptor (A1), heparin, and collagen (A1 and A3). The A2 domain contains the cleavage site for *ADAMTS13*, which regulates the multimer size of VWF [12,13,14,15]. VWF’s conformation in plasma prevents premature receptor binding, but shear stress exposes its GPIbα binding site, enhancing platelet adhesion [12].

Beyond hemostasis, VWF plays a role in angiogenesis through interactions with VEGFR-2 and integrin αvβ3, activating signaling pathways related to gene expression [12]. In the bloodstream, VWF promotes platelet adhesion to damaged vessels and enhances aggregation through glycoprotein IIb/IIIa receptors, stabilizing the clot. It also protects factor VIII from degradation, ensuring effective clot formation at injury sites [12,15,16].

#### 3.1.2. Role in Primary and Secondary Hemostasis

Primary hemostasis is the initial response to blood vessel injury, aimed at forming a temporary platelet plug to prevent blood loss. A key player in this process is VWF, which is stored in Weibel–Palade bodies within endothelial cells and platelets [17]. Upon vessel injury, exposed collagen triggers platelet adhesion, with VWF bridging the gap between the damaged vessel wall and circulating platelets. VWF binds to collagen and the GPIb receptor on platelets, anchoring them to the site of injury. Once adhered, platelets activate, change shape, and extend filopodia to enhance their interactions [18,19]. They release granules containing mediators that further promote the activation and recruitment of additional platelets [19,20]. VWF also binds to platelet glycoprotein IIb/IIIa receptors, increasing their affinity for fibrinogen and facilitating platelet aggregation to form a stable plug [17].

In secondary hemostasis, VWF serves as a carrier for coagulation factor VIII, protecting it from premature degradation [21]. By binding to the exposed subendothelial matrix, VWF promotes the accumulation of factor VIII, which is crucial for effective coagulation. Activated factor VIIIa enhances factor IXa activity, facilitating the conversion of factor X to Xa and ultimately generating thrombin and fibrin. This process reinforces the platelet plug, with VWF stabilizing factor VIII and enhancing its interactions with other coagulation factors, although it does not directly participate in the coagulation cascade [21,22].

#### 3.1.3. Genetic Basis of Congenital VWD

VWD is the most common autosomal inherited bleeding disorder, affecting approximately 1% of the general population [23,24]. It arises from a quantitative and/or qualitative deficiency of VWF, located on chromosome 12 [23,25]. There are three types of VWD. Type 1 is the most common, accounting for 60–70% of cases [26], characterized by a partial reduction in VWF levels and activity [27]. Recent studies show mutations in about 90% of patients, primarily heterozygous missense mutations [28]. Type 2 involves qualitative defects in VWF, with normal or elevated levels but impaired function. It includes subtypes such as Type 2A, which is characterized by reduced high molecular weight multimers (HMWM), impairing platelet adhesion; Type 2B, with increased VWF affinity for platelets, leading to spontaneous aggregation and bleeding; Type 2M, with reduced binding to platelets without loss of high molecular weight multimers; Type 2N, with defects in VWF binding to factor VIII, mimicking hemophilia A [27]. Type 3 is the most severe form, characterized by undetectable VWF levels, resulting from null alleles and leading to severe bleeding [29].

Additionally, mutations in the *ADAMTS13* gene can mimic or exacerbate VWD symptoms, particularly in Type 2A [30,31]. Mutations in the *F8* gene, related to factor VIII, are associated with Type 2N VWD [32]. Individuals with non-0 blood groups (A, B, or AB) have a higher risk of venous thromboembolism due to elevated levels of factor VIII and VWF, with about 66% of the variation in VWF plasma levels attributed to genetic factors, including the ABO blood group, which influences VWF breakdown [33].

#### 3.1.4. Causes and Mechanisms of AVWD

AVWD is a rare condition characterized by a deficiency or dysfunction of VWF due to secondary factors that disrupt its normal levels or function, leading to significant heterogeneity. AVWD is commonly associated with conditions such as monoclonal gammopathy, myeloproliferative and malignant disorders, autoimmune diseases, aortic valve stenosis, and the presence of left ventricular assist devices [34,35,36]. 

The mechanisms underlying AVWD vary by disorder and may include reduced VWF synthesis, formation of autoantibodies, mechanical destruction of VWF from high shear stress, and increased proteolysis [35,36,37]. Autoantibodies can form immune complexes with VWF, leading to accelerated clearance and decreased functional levels [36]. Endocrine disorders like hypothyroidism can decrease VWF synthesis due to the effects of thyroid hormones on endothelial cells [38]. Additionally, malignant cell clones may absorb high molecular weight multimers, reducing their availability [39]. Myeloproliferative disorders can paradoxically increase bleeding risk due to heightened platelet counts, which can cause increased shear stress and proteolysis of VWF. Certain medications, including hydroxyethyl starch, ciprofloxacin, and valproic acid, have also been linked to AVWD; discontinuation of these drugs often normalizes hemostatic parameters [36]. The classification, diagnosis, and treatment of AVWD depend on the underlying condition or causative factor [40].

#### 3.1.5. Interrelationship between Aortic Stenosis and VWF Degradation

**Figure 3:**
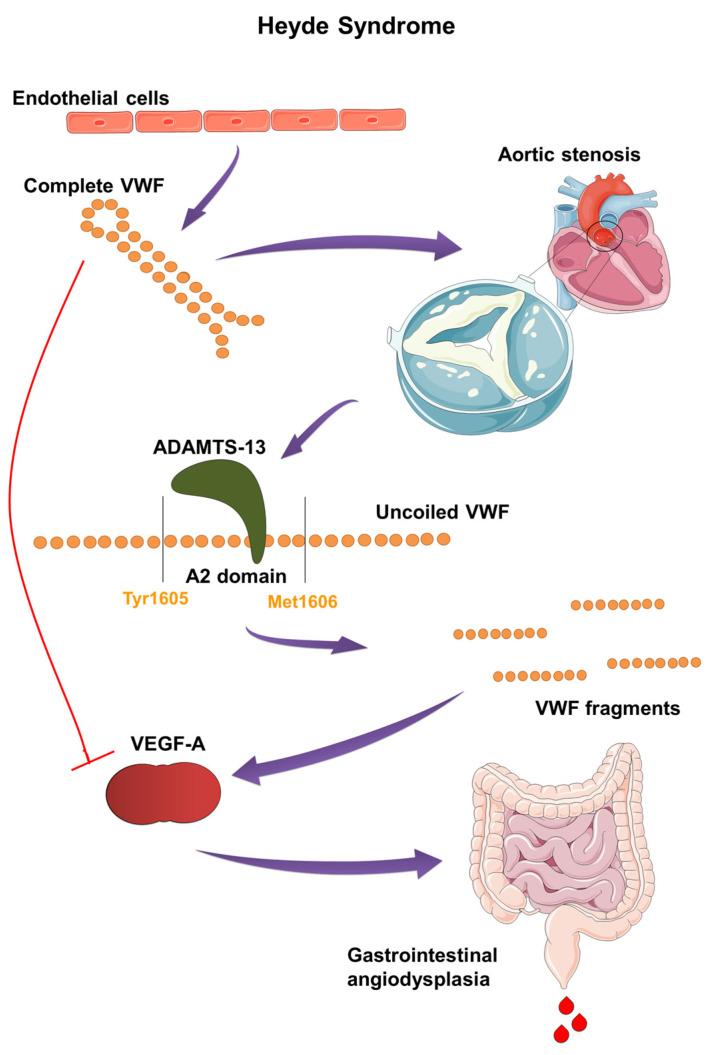
Pathogenesis and molecular mechanism of Heyde syndrome: The narrowed aortic valve increases shear stress on VWF, causing its multimers to uncoil and elongate. This exposes the A2 domain, making VWF susceptible to cleavage by *ADAMTS13* enzyme. The resulting smaller VWF fragments lose their hemostatic function and fail to inhibit VEGF. The loss of VEGF inhibition leads to abnormal blood vessel formation, or angiodysplasia, particularly in the gastrointestinal tract, causing bleeding.

The exceptionally large size of VWF multimers makes them particularly vulnerable to physical forces [36]. More recently, AVWD has been frequently diagnosed in patients with aortic valve stenosis [41]. In aortic stenosis, the pathophysiology of AVWD is complexly linked to the effects of shear stress on VWF caused by the hemodynamic changes associated with a narrowed aortic valve [42]. Shear stress forces are essential in regulating VWF’s hemostatic activity. As shear rates increase and flow accelerates, VWF uncoils, and its extended multimers can reach lengths of approximately 1300 nm. These uncoiled multimers may form fibrils through end-to-end self-association, which can extend for hundreds of microns [35]. In patients with aortic stenosis, the constricted valve causes high-velocity blood flow, increasing shear stress on blood components. The high shear environment causes VWF multimers, particularly the HMWM, to unfold and elongate, exposing the A2 domain [43]. The exposed A2 domain then serves as a substrate for *ADAMTS13* by revealing the scissile bond Tyr1605-Met1606, which is crucial for the enzyme’s cleavage activity [31,35]. Elevated shear stress accelerates *ADAMTS13*-mediated proteolysis, leading to a significant reduction in HMWM size, diminishing their hemostatic activity (Figure 3) [42]. In vitro studies show that unfolding of the A2 domain occurs in less than 1 s. Furthermore, in vivo, this unfolding and cleavage can occur within 200 s in response to sudden changes in shear stress [44]. Additionally, shear stress induces conformational changes in VWF, unveiling a cryptic binding site for GPIbα within the A1 domain, which enhances VWF’s interaction with platelets. High shear rates and platelet binding both increase VWF’s sensitivity to cleavage by *ADAMTS13*. Mutations that enhance VWF binding to GPIbα, such as those seen in VWD-type 2B, are associated with increased proteolysis [45]. AVWD in this context typically resembles type 2A VWD, where the quantity of VWF may be normal, but its functionality is compromised [46]. Symptoms such as easy bruising, mucosal bleeding, and gastrointestinal bleeding often correlate with the severity of aortic stenosis and VWF abnormalities [42,47].

Vincentelli et al. showed that VWF abnormalities occur not only in severe but also in moderate aortic stenosis. In aortic stenosis patients, 92% of severe and 50% of moderate cases had prolonged closure times under high shear stress, with decreased collagen-binding activity and reduced HMWM in both groups. VWF abnormalities were correlated with transvalvular gradient and stenosis-induced shear stress, directly reflecting AS severity [48]. Tamura et al. also demonstrated that the VWF multimer ratio and the VWF multimer index (the ratio of a patient’s VWF multimer ratio to that of a control) were inversely correlated with the peak aortic gradient, reflecting the severity of aortic stenosis [49]. Kellermair et al. analyzed the role of HMWM ratios in differentiating true-severe from pseudo-severe low-flow, low-gradient aortic stenosis. In 83 patients, HMWM ratios were significantly lower in true-severe compared to pseudo-severe aortic stenosis. The multimer ratio correlated strongly with transvalvular pressure gradients and showed increased degradation during dobutamine stress echocardiography. This study concluded that the HMWM ratio is a valuable biomarker for subclassifying aortic stenosis and can identify true-severe aortic stenosis without other imaging techniques [50]. Van Belle et al. described a “series circuit” model, in which, in stenotic heart valve disease, HMWM degradation occurs when a large portion of the blood is subjected to high shear stress, as seen in valve stenosis. As shear stress at the valve increases (indicated by a higher transvalvular gradient), the amount of HMWM in the peripheral blood decreases. This relationship between HMWM loss and the transvalvular gradient helps accurately quantify the severity of heart valve disease [10]. 

However, after surgical interventions like surgical aortic valve replacement (SAVR) or TAVR, many patients see a normalization of VWF levels and function, indicating that correcting the hemodynamic disturbance can restore normal hemostasis and resolve the AVWD [42,51]. Shear stress-induced degradation of VWF is also observed in other cardiac conditions, including left ventricular assist devices, hypertrophic cardiomyopathy, and mitral and aortic regurgitation. Furthermore, the degradation of VWF and the development of AVWD have been reported in cases of dysfunction of both surgical and transcatheter aortic prostheses [5].

### 3.2. Understanding Angiodysplasia and Its Implications in Heyde Syndrome

#### 3.2.1. Overview of Angiodysplasia

Angiodysplasia, also called angioectasia or gastrointestinal angiodysplasia, represents an abnormal small blood vessel in the mucosal and submucosal layers of the gut wall. The small blood vessel is tortuous, dilated, and contains minimal amounts of smooth muscle fibers [52]. Although a relatively rare endoscopic finding, this acquired abnormality is the most common vascular abnormality in the gut [53]. Angiodysplasia is most commonly found in the colon during routine endoscopic examinations, specifically in the ascending colon and the cecum, although it can be found in any part of the gastrointestinal tract [54]. However, with the development of capsule endoscopy, the actual incidence of angiodysplasia has been better studied, and it is now known that the most common location of angiodysplasia is in the proximal part of the small bowel (up to 80% of examined patients), with the colon being the second most frequent location, followed by the stomach [55]. It is also common for more than one site of angiodysplasia to be found, with up to 60% of examined patients showing lesions in more than one segment of the gastrointestinal tract [56]. 

The prevalence of colonic angiodysplasia in healthy, asymptomatic individuals is very low (0.83%); the lesions are smaller than 10 mm, and the natural course of the condition is benign, with a three-year bleeding rate of 0% per one study [57]. Small-bowel angiodysplasia mostly has the same course, with no negative impact on patient survival [58]. The presence of a dilated blood vessel in the mucosa of the digestive tract can lead to gastrointestinal bleeding, which is a common occurrence in angiodysplasias, either from the upper or lower gastrointestinal tract [59,60]. Angiodysplasias are the most common cause of occult bleeding from the small bowel in older patients [61].

#### 3.2.2. Etiology and Mechanisms of Angiodysplasia Formation

Angiodysplasias are an acquired abnormality whose exact etiology remains not well understood. Angiogenesis represents a crucial mechanism in the development of angiodysplasias. Increased angiogenesis by upregulation of the most potent proangiogenic factor, vascular endothelial growth factor (VEGF), is responsible for the development of angiodysplasia [62]. Basic fibroblast growth factor (bFGF) also plays a significant role in angiogenesis [63]. Angiogenesis, the process of forming new blood vessels from existing ones, occurs physiologically in wound healing and the menstrual cycle. Dysregulation of angiogenesis can contribute to various diseases, including diabetes, cancer, and macular degeneration [64]. 

Key angiogenic factors, such as VEGF and angiopoietins (Ang), play crucial roles in this process. VEGF-A, a primary regulator of angiogenesis, acts through VEGFR-2, a tyrosine kinase receptor, to stimulate endothelial cell proliferation, migration, and the formation of new vessel sprouts. Meanwhile, Ang-1 and Ang-2, which interact with the Tie-2 receptor on endothelial cells, are critical for the later stages of vessel formation. Ang-1 promotes the stabilization and integrity of the vascular network, while Ang-2 contributes to vessel destabilization and regression [65]. In patients with angiodysplasia, a study plagued by a small sample size, which reduced its statistical significance, showed that VEGF levels in serum are much higher than in healthy controls, while another study found that both VEGF and bFGF have been found in the endothelial lining of colonic blood vessels with angiodysplasia, in contrast to healthy colon endothelium, where there were none [66,67]. The cause of expression of proangiogenic factors in the endothelium is not certain, with the most cited hypothesis being increased contractility of the muscularis propria in the gut, which leads to intermittent obstruction of the submucosal veins, followed by congestion of the capillaries and the formation of arteriovenous collaterals [68,69].

#### 3.2.3. Risk Factors for Angiodysplasia

There are certain risk factors associated with angiodysplasias: age (>60 years), chronic obstructive pulmonary disease, aortic stenosis (Heyde syndrome) [70], left ventricular assist devices, vWD, venous thromboembolism, ischemic heart disease, liver cirrhosis, and renal failure [2,71]. These risk factors share some common denominators: chronic obstructive pulmonary disease, ischemic heart disease, and aortic stenosis all lead to tissue hypoxia, which favors angiogenesis [2,72]. Other risk factors, like vWD, favor angiogenesis because endothelial VWF is an angiogenesis regulator, and its deficiency leads to enhanced VEGF-dependent angiogenesis [73]. Angiodysplasias are very common in patients with chronic renal failure, while also being a leading cause of gastrointestinal bleeding in these patients, causing up to 30% of lower gastrointestinal bleeding episodes, compared to only 5–6% in the general population [57]. While the cause of high angiodysplasia incidence in this group of patients is unclear, the increased probability of bleeding is due to platelet dysfunction caused by chronic renal failure [74]. Mechanisms responsible for platelet dysfunctions include the release of toxins and nitric oxide, as well as a reduced amount of agonists like serotonin, adenosine diphosphate, and thrombin, which are important for platelet function [75,76].

#### 3.2.4. Angiodysplasia in Heyde Syndrome

The association between aortic stenosis and gastrointestinal bleeding was first reported by Heyde et al. in 1958 [1]. Later, Rogers et al. in 1980 suggested an association with gastrointestinal angiodysplasia [77]. Subsequent studies revealed that aortic stenosis is linked to a significantly higher incidence of occult gastrointestinal bleeding compared to other valvular diseases [78,79]. Surgical treatment of aortic stenosis has been shown to reduce gastrointestinal bleeding over long periods, often for years or even decades [80,81]. However, some controversy exists due to methodological flaws in these studies, with other research failing to replicate the correlation but finding links between aortic sclerosis and gastrointestinal bleeding [82,83,84]. A more recent study found that 32% of elderly patients with angiodysplasia confirmed via endoscopy also had aortic stenosis, a significantly higher prevalence than in the control group [85]. 

VWF secreted by endothelial cells is believed to modulate VEGFR signaling through integrin αvβ3, influencing both the promotion and inhibition of angiogenesis [62,65]. While early research suggested that integrin αvβ3 had a solely pro-angiogenic role, more recent studies indicate that it can exhibit both pro- and anti-angiogenic effects depending on the extracellular environment and specific ligands involved [86]. VWF interacts with αvβ3 integrin in endothelial cells to inhibit angiogenesis through extracellular and intracellular pathways [12,87]. By binding to integrin αvβ3 via its C-terminal RGD motif, VWF stabilizes integrin expression on the endothelial surface [88]. This binding reduces VEGFR signaling and activity, while low VWF levels lead to increased VEGF activity and signaling [62]. VWF is also crucial for forming Weibel–Palade bodies, which store Ang-2. By promoting Ang-2 storage and inhibiting its synthesis, VWF regulates Ang-2 levels. Upon endothelial cell activation, Ang-2 is released, enhancing VEGFR-2 signaling, destabilizing blood vessels, and promoting angiogenesis (Figure 3) [12]. Additionally, VWF impacts vascular maturation by affecting αvβ3 expression in vascular smooth muscle cells (VSMC) during vascular development. The binding of VWF to αvβ3 on VSMCs is essential for their recruitment and arterial maturation, so a loss of VWF disrupts blood vessel formation through various mechanisms impacting both endothelial cells and VSMCs [12]. 

The leading hypothesis posits that AVWD, due to HMWM deficiency secondary to aortic stenosis, is a primary risk factor for the development of angiodysplasia in the gut [85]. The narrowed aortic valve in aortic stenosis causes turbulent blood flow and high shear stress, which damages VWF by fragmenting it, particularly the HMWM, reducing its platelet-binding and clot-forming abilities. The high shear conditions also impair VWF’s functional conformation and accelerate its degradation by *ADAMTS-13* protease. These disruptions lead to diminished clotting function and increased bleeding tendencies in aortic stenosis patients. In Heyde syndrome, angiodysplastic vessels create higher shear stress than normal vessels, and the lack of functional VWF in aortic stenosis patients increases their risk of gastrointestinal bleeding [89]. Despite the presence of angiodysplasia, gastrointestinal bleeding often resolves after aortic valve replacement, indicating that bleeding is more likely related to shear stress-induced loss of HMWM VWF rather than the angiodysplasia itself. Hence, aortic valve replacement typically resolves gastrointestinal bleeding in these patients [62].

### 3.3. Clinical Approach and Diagnosis

#### 3.3.1. History and Physical Examination

For a comprehensive evaluation of a patient with suspected Heyde syndrome, the history and physical examination should focus on identifying aortic stenosis, gastrointestinal bleeding, and signs of impaired hemostasis indicative of AVWD. Symptoms associated with aortic valve disease include dyspnea on exertion, syncope, fatigue, and exertional angina. The physical examination should include a detailed cardiac assessment, particularly noting features of aortic stenosis such as a harsh, late-peaking systolic murmur, most prominent over the second right intercostal space and radiating to the carotid arteries. Additional findings may include a slow and delayed carotid upstroke, a sustained point of maximal impulse, and a reduced or absent aortic second sound [90].

Patients with gastrointestinal bleeding can exhibit a range of symptoms, from asymptomatic occult bleeding to acute manifestations like hematemesis, hematochezia, and melena. A digital rectal examination may reveal fresh blood or dark stool indicative of melena [91]. In Heyde syndrome, the bleeding is typically painless, chronic, or recurrent, and rarely leads to acute hemorrhagic shock [92]. Symptoms of anemia, such as fatigue, shortness of breath, palpitations, and pallor, may also be present [93]. Indicators of AVWD include easy bruising, mucosal bleeding, hemarthrosis, and hematomas [92].

#### 3.3.2. Laboratory and Imaging Assessments

The initial laboratory workup should encompass a complete blood count along with biochemical and coagulation parameters. Low levels of Hb, Hct, MCV, serum iron, and ferritin are indicative of iron deficiency anemia due to chronic blood loss [94]. A sudden drop in hemoglobin may suggest acute bleeding [91]. In Heyde syndrome, platelet counts and coagulation parameters, such as PT and PTT, are usually within normal ranges, although prolonged PTT may be observed as a result of low factor VIII levels [92]. The fecal occult blood test may be positive in cases of occult bleeding [91], and elevated levels of pro-BNP can indicate aortic stenosis with heart failure [95].

In patients with type 2 VWD, the levels of VWF antigen and VWF:Rco activity are typically within normal ranges. However, a VWF:Rco/VWF antigen ratio of less than 0.7 serves as a reliable diagnostic marker for AVWD type 2A. Gel electrophoresis, which quantifies HMWM, is considered the gold standard for diagnosing VWD associated with aortic valve stenosis. Despite its accuracy, this method is time-consuming and costly. In contrast, the PFA offers a more straightforward, point-of-care screening test that measures platelet aggregation under shear stress, providing a sensitive method for detecting VWD [62,92].

Transthoracic echocardiography is the standard tool for assessing aortic stenosis. It is essential to measure the AV Vmax, mean gradient, and AVA in all patients undergoing evaluation for aortic valve stenosis to accurately describe the severity of the condition [96].

Upper and lower endoscopy are the primary diagnostic tools for angiodysplasia, with capsule endoscopy serving as an advanced and efficient option in detecting small bowel angiodysplasias. Capsule endoscopy is considered superior to small bowel radiography, push enteroscopy, computed tomography, and angiography [2,97].

For a comprehensive differential diagnosis in patients with aortic stenosis, it is crucial to exclude other potential causes of gastrointestinal bleeding. These include peptic ulcer disease, the use of nonsteroidal anti-inflammatory drugs, esophageal varices, and anticoagulant use. Additionally, nutritional deficiencies such as celiac disease and the presence of malignancies should also be considered [92,94].

### 3.4. Therapeutic Strategies

#### 3.4.1. Aortic Stenosis Management

Aortic valve replacement, either through SAVR or TAVR, is widely recognized as an effective treatment for patients with Heyde syndrome [92,98,99]. In recent years, TAVR has become the preferred treatment for patients with aortic stenosis who are at higher surgical risk [100]. After valve replacement, VWF levels increase rapidly, with full recovery observed in 86% of patients within 24 h. Maximum recovery is achieved within 3 days after SAVR in 95% of patients and within 3–7 days after TAVR in 91% of patients. The cessation of VWF proteolysis post-AVR leads to rapid recovery from AVWD due to the release of stored VWF, facilitated by the shift from pathological to physiological shear stress and by endothelial repair. Complete cessation of gastrointestinal bleeding is observed in 82% of patients after SAVR and 64% after TAVR. The outcomes of both SAVR and TAVR surpass those of conventional treatment options, prompting consideration of valve replacement solely for gastrointestinal bleeding management in Heyde syndrome [98]. 

In the search for new, minimally invasive alternatives to improve safety and patient outcomes, lateral minithoracotomy endoscopic robotic aortic valve replacement (RAVR) has been developed. An initial international multicenter study involving 200 cases demonstrated that, in centers with established robotic expertise, RAVR is a reproducible and safe procedure, yielding excellent early results [101]. However, many patients, such as those with severe comorbidities, limited life expectancy, or those who decline invasive treatment—as was the case with our patient—are considered unsuitable for such interventions. Therefore, a prospective, multicenter, single-arm study involving 40 adult patients with severe symptomatic aortic stenosis evaluated the safety and efficacy of a novel non-invasive transthoracic ultrasound therapy as an alternative treatment. After six months, this therapy was shown to be both safe and feasible, demonstrating promise in improving valvular function by softening calcified valve tissue [102]. Additionally, several drugs are under investigation as potential options for slowing the progression of aortic stenosis, including evolocumab and dipeptidyl peptidase-4 inhibitors [100]. 

#### 3.4.2. Management of Gastrointestinal Hemorrhage

The initial approach to gastrointestinal bleeding in suspected Heyde syndrome involves stabilizing the patient by administering intravenous fluids and blood transfusions before identifying the source of bleeding or performing therapeutic interventions [92]. Different endoscopic techniques are used, with APC being the most commonly employed method. Other endoscopic methods for hemostatic treatment include monopolar electrocoagulation, bipolar electrocoagulation, photocoagulation, and Argon laser. In cases where endoscopic therapy is insufficient, selective embolization via angiography can be employed; it is frequently used for actively bleeding small-bowel angiodysplasias due to their inaccessibility [2]. Device-assisted enteroscopy, including double-balloon enteroscopy, is commonly utilized for the diagnosis and treatment of small-bowel angiodysplasias, typically after initial evaluation with capsule endoscopy. Spiral enteroscopy is an emerging technology that shows promise, offering significantly reduced procedural times compared to double-balloon enteroscopy. However, further studies are needed to fully assess its safety and feasibility [103].

Several pharmacologic agents are used as prophylactic therapies for angiodysplasia. Somatostatin analogs, such as octreotide and lanreotide, increase vascular resistance, reduce duodenal and splanchnic blood flow, and inhibit angiogenesis [2,97]. A systematic review and meta-analysis of 11 studies (one randomized controlled trial and ten cohort studies) involving 212 patients demonstrated that somatostatin analogs reduced transfusion needs with an incidence rate ratio of 0.18, resulting in an average decrease of 10.5 transfusions over 12 months. Approximately 83% of patients achieved at least a 50% reduction in transfusion requirements. The therapy was more effective for angiodysplasias in the small bowel and colon, with octreotide showing greater efficacy than lanreotide. Adverse events occurred in 18% of patients, with 5% discontinuing therapy. Overall, the treatment was deemed safe and effective [104].

In a separate randomized trial, octreotide significantly reduced the need for transfusions and endoscopies in patients with gastrointestinal angiodysplasias and transfusion-dependent anemia compared to standard care. This trial included 62 patients, with those in the octreotide group receiving 40 mg intramuscularly every 28 days, while the control group received transfusions of iron or blood as needed over a 1-year period. Patients treated with octreotide required 10 fewer transfusions and about one fewer endoscopy per patient. Additionally, 61% of patients receiving octreotide experienced a ≥ 50% reduction in transfusion needs, compared to only 19% in the usual care group. Adverse events, such as diarrhea and injection site pain, were rare, leading to discontinuation in only 6% of patients [105].

Another agent, thalidomide, works by inhibiting VEGF and subsequent angiogenesis, making it effective in treating gastrointestinal angiodysplasias [2,93]. It is typically administered orally at doses of 50 to 300 mg for 1 to 6 months, with effects lasting even after discontinuation. Studies, including randomized controlled trials, have shown that thalidomide significantly reduces the need for blood transfusions and increases hemoglobin levels, particularly as a second- or third-line therapy after endoscopy or somatostatin analogs [106]. In a multicenter, placebo-controlled trial involving 150 patients with recurrent bleeding, 68.6% of those receiving 100 mg of thalidomide daily for four months, and 51.0% of those on 50 mg daily for four months, achieved at least a 50% reduction in bleeding episodes, compared to 16.0% in the placebo group after one year of follow-up. In this study, adverse events were commonly reported in patients receiving thalidomide, with occurrence rates of 68.6% for the 100 mg dose and 55.1% for the 50 mg dose. Around 3% of patients discontinued treatment due to these adverse events. Reported side effects included somnolence, peripheral edema, elevated liver enzyme levels, and dizziness [107]. However, based on thalidomide’s use in multiple myeloma, long-term use, particularly at higher doses or in older patients, has been associated with increased side effects such as neutropenia, bradycardia, deep-vein thrombosis, and neurological issues like peripheral neuropathy and tremors [108].

Bevacizumab is a recombinant humanized monoclonal antibody that inhibits VEGF by directly binding to the protein, which suppresses angiogenesis [97]. Primarily used in cancer treatment to inhibit tumor growth and metastasis, bevacizumab has also been employed to manage bleeding in conditions like hereditary hemorrhagic telangiectasia and gastric antral vascular ectasia. Although bevacizumab is promising for gastrointestinal angiodysplasias, data remain limited. Administered at lower doses than in oncology, bevacizumab has shown favorable results but is considered a last-resort option due to safety concerns and high cost [109]. Further randomized trials are necessary to determine the definitive roles of these agents in managing angiodysplasia. Surgery remains a viable rescue option for acute or recurrent bleeding that does not respond to initial measures [2,97].

#### 3.4.3. Treatment of AVWD

The management of AVWD in Heyde syndrome involves two primary strategies: enhancing the endogenous release of VWF or using VWF-containing treatments. Desmopressin rapidly stimulates the release of VWF and factor VIII from endothelial cells, achieving this effect within 1–2 h. Monitoring VWF and factor VIII levels is essential to prevent tachyphylaxis. VWF-containing treatments include fresh frozen plasma, cryoprecipitate, and virally inactivated VWF/FVIII concentrates. These options address VWF deficiency but carry risks such as thromboembolic events and infections [62]. Vonvendi, a novel recombinant VWF product, offers increased activity without *ADAMTS13*-mediated degradation, which may reduce the risk of thrombosis by eliminating the need for concurrent factor VIII administration [62,110].

Lysine analogs, such as epsilon-aminocaproic acid, are antifibrinolytic agents that inhibit plasminogen to prevent clot dissolution. Tranexamic acid, another effective antifibrinolytic, binds to plasminogen and can be administered in combination with desmopressin or VWF-containing concentrates [62].

#### 3.4.4. Patient Management and Follow-Up: Our Strategy and Insights from Current Literature 

SAVR and TAVR are cornerstones in the management of Heyde syndrome, significantly surpassing the effectiveness of conventional treatment methods [98]. A significant long-term study by King et al. (1987) followed three patient groups over a period of 8 to 12 years. Among the 40 patients who did not undergo abdominal or aortic valve surgery, all continued to experience persistent bleeding and chronic anemia, necessitating ongoing blood transfusions. Furthermore, among the thirty-seven patients who underwent abdominal surgery for bleeding, only two remained free of postoperative bleeding. In contrast, of the sixteen patients who underwent aortic valve replacement, only one experienced postoperative bleeding, which was attributed to elevated prothrombin time [111].

In a systematic review by Saha et al., the recurrence of bleeding in patients with Heyde syndrome after SAVR was reported at 5.3%, while the recurrence rate after TAVR was 10.5%. In stark contrast, the recurrence of bleeding in patients who did not receive valve replacement was alarmingly high at 50%. These findings highlight the efficacy of valve replacement in preventing further bleeding complications in patients with Heyde syndrome [9].

However, our patient declined the proposed treatment, necessitating the exploration of alternative options. These may include endoscopic interventions to address gastrointestinal bleeding from angiodysplasia, along with a multimodal conservative management approach. Firstly, we focused on correcting the anemia with transfusion of concentrated RBC and with iron supplementation. To manage heart failure associated with aortic stenosis and probable anemia, we administered diuretics during the patient’s hospitalization and prescribed standard cardiological therapy at discharge. For the treatment of colon angiodysplasia, we selected APC, which is the most commonly used and preferred method [2]. Although APC may have inferior effectiveness in preventing bleeding in individuals with Heyde syndrome, we believe these findings could be influenced by the heterogeneity of the study population and the small sample size [9]. Therefore, we will await more comprehensive randomized studies before drawing definitive conclusions, while continuing to adhere to established knowledge in the meantime. 

Somatostatin analogs present a reasonable treatment option for gastrointestinal bleeding due to their efficacy and favorable safety profile, though their high cost and limited availability pose challenges [106,109]. In a case report by Cheema et al., intramuscular octreotide at a dosage of 30 mg monthly effectively restored hemoglobin levels without recurrence of bleeding in a patient with Heyde syndrome, outperforming intravenous octreotide [112]. 

Thalidomide is another potentially effective agent, but its adverse effects, particularly in elderly patients [106,109], like in our case, limit its use. Hvid-Jensen et al. reported on a patient with severe gastrointestinal bleeding due to hypertrophic subvalvular obstructive cardiomyopathy, for whom endoscopic procedures were ineffective. The patient was successfully treated with a combination of oral thalidomide (50 mg) and intramuscular octreotide (20 mg monthly), leading to anemia recovery within three months and enabling transcoronary ethanol ablation [113].

Bevacizumab is an emerging treatment option, though its evidence base remains limited, and its cost is high [109]. In a case report by Song et al., systemic bevacizumab therapy (5 mg/kg biweekly for two months, followed by monthly maintenance doses) effectively treated severe bleeding in a Heyde syndrome patient unresponsive to endoscopic procedures, tranexamic acid, and octreotide [114]. A pilot study assessing intravenous bevacizumab in five LVAD patients with refractory gastrointestinal bleeding due to angiodysplasias showed significant reductions in blood transfusions, hospitalizations, and scoping procedures. The treatment was well-tolerated without serious side effects, indicating bevacizumab as a promising option for managing refractory gastrointestinal bleeding, although further studies are needed to establish its long-term efficacy and safety [115].

Regarding the treatment of AVWD in Heyde syndrome, tranexamic acid may be a possible option for managing chronic bleeding due to angiodysplasias. Grooteman et al. administered 1000 mg of tranexamic acid daily after unsuccessful treatment with octreotide and thalidomide in a patient with chronic recurrent bleeding, which resulted in stabilized hemoglobin levels and a reduced need for transfusions [116].

**Figure 4:**
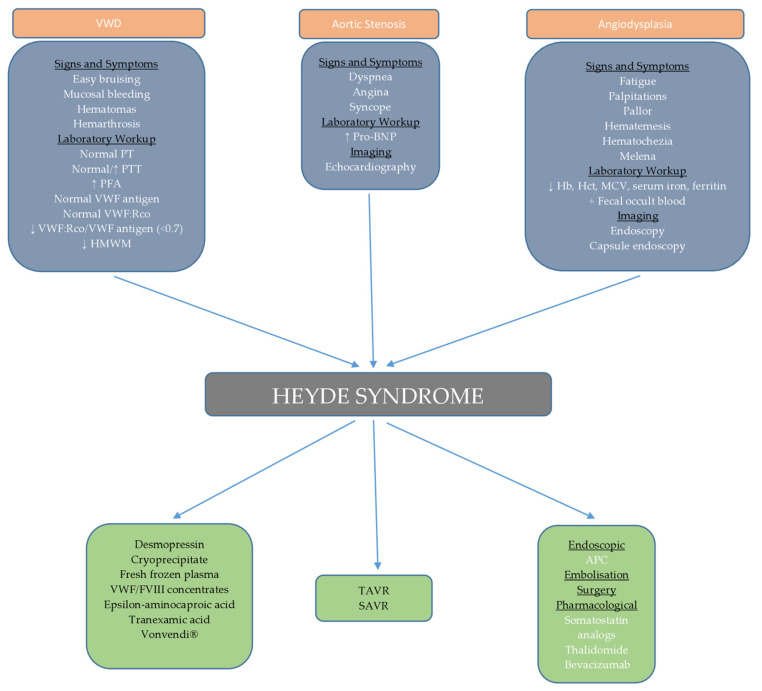
Schematic representation of diagnostic and therapeutic approach to Heyde syndrome.

In our case, we organized regular monitoring of the patient’s clinical and laboratory parameters through monthly check-ups. Regular endoscopic and echocardiographic evaluations are also planned. We opted for a “watch and wait” approach due to the unavailability of certain therapeutic modalities and the potential side effects of treatment, considering the patient’s age. In the event of bleeding or a drop in hemoglobin, our first-line treatment will be endoscopic intervention along with intramuscular octreotide injections at a dose of 40 mg monthly, as supported by randomized trial [105], along with supportive measures such as blood and iron supplementation. If this treatment proves ineffective, additional rescue therapies, such as the addition of thalidomide or tranexamic acid, will be considered, with careful monitoring for side effects. This case report provides an in-depth review of the current understanding of the pathogenesis, diagnosis, and treatment options for Heyde syndrome, an area still under active research. Managing patients with Heyde syndrome, especially those with multiple comorbidities, highlights the need for a personalized, multidisciplinary approach. By sharing this case, we aim to offer clinicians a practical framework for decision-making in complex cases where therapeutic options are limited or carry significant risks. Through this case and the accompanying diagnostic and therapeutic algorithm (Figure 4), we hope to provide a valuable resource for clinicians while contributing to further research in the field of Heyde syndrome management.

### 3.5. Future Perspectives

To enhance the understanding and treatment of Heyde syndrome, future research should focus on several key areas. Firstly, conducting population studies is essential to accurately determine the incidence of Heyde syndrome, especially among the elderly, where aortic stenosis is a prevalent condition.

Investigating the pathogenesis of the syndrome, including developing representative in vivo models, will provide valuable insights and aid in identifying new therapeutic targets. Developing affordable, widely accessible, and rapid tests for assessing VWD is crucial for improving diagnostic accuracy. Additionally, identifying new biomarkers, such as Ang-2 [117], for detecting small bowel angiodysplasias is vital for facilitating quicker diagnosis and better patient outcomes.

The integration of artificial intelligence (AI) into the early diagnosis and treatment of conditions like Heyde syndrome holds significant potential for advancing the field. Cohen-Shelly et al. developed an AI-enabled electrocardiogram that effectively detects moderate to severe aortic stenosis and serves as a valuable screening tool [118]. Holste et al. furthered AI’s role in aortic stenosis diagnosis by creating and externally validating a novel deep learning model that relies on single-view 2D echocardiography, eliminating the need for Doppler imaging. This model holds promise for point-of-care screening, offering a practical solution for the early detection of aortic stenosis [119]. Beyond diagnostics, AI shows potential in guiding intra-procedural operations during TAVR by assisting with valve sizing and procedural navigation through real-time segmentation of heart valves and delivery systems, thus improving outcomes [120]. Chu et al. demonstrated AI’s effectiveness in identifying angiodysplasia lesions [121], while Laat-Kremers et al. developed a neural network with over 90% sensitivity in diagnosing antiphospholipid syndrome [122], underscoring AI’s growing utility in the accurate diagnosis of complex conditions, including coagulopathies. These advancements highlight the transformative role AI can play across various aspects of healthcare, from diagnosis to treatment, offering more accurate, efficient, and personalized care.

Randomized studies comparing various treatment options, including SAVR and TAVR, as well as different pharmacological agents, are necessary to identify the most effective treatments. Incorporating Heyde syndrome into clinical guidelines will help standardize and improve treatment practices.

Collectively, these efforts will advance the diagnosis, treatment, and understanding of Heyde syndrome, ultimately leading to improved patient outcomes.

## 4. Conclusions

In our case, the patient was promptly diagnosed and successfully treated with argon plasma coagulation (APC). Although the patient refused aortic valve reconstruction, which is currently the only curative treatment for long-term management, a continuous follow-up plan has been established, including potential endoscopic and pharmacological interventions in the future.

Understanding the molecular mechanisms involved in Heyde syndrome, the main focus of this review, is crucial for improving diagnostic methods and advancing therapeutic strategies. Future research should prioritize accurate epidemiological studies, further elucidation of molecular mechanisms, the development of efficient and cost-effective diagnostic tests, and the creation of new imaging and treatment modalities. Additionally, comparative studies on the currently available treatment options are essential.

## Figures and Tables

**Table 1:** Laboratory data include initial results at the time of hospitalization, at discharge, and at a follow-up appointment two months after discharge from the hospital.

Variable	Initial (Day 0)	At Discharge (Day 5)	Two Months after Discharge	Reference Range, Adults
Red Blood Cells (RBC)	2.93 × 10^12^/L	3.48 × 10^12^/L	4.4 × 10^12^/L	4.34–5.72 × 10^12^/L
Platelets	161 × 10^9^/L	176 × 10^9^/L	251 × 10^9^/L	150–450 × 10^9^/L
Haemoglobin (Hb)	60 g/L	86 g/L	112 g/L	138–175 g/L
Mean Cell Volume (MCV)	72 fL	75 fL	85 fL	83.0–100.0 fL
Serum Iron	2.1 µmol/L	6 µmol/L	17 µmol/L	14–32 µmol/l
Total Iron-Binding Capacity (TIBC)	75.1 µmol/L	73 µmol/L	65 µmol/L	49–72 µmol/l
Unsaturated Iron Binding Capacity (UIBC)	77 µmol/L	75 µmol/L	70 µmol/L	23.45–76.08 µmol/L
Ferritin	11.6 ng/mL	15 ng/mL	21 ng/mL	30–400 ng/mL
NT-proBNP	3352 pg/ml	/	679 pg/ml	150 pg/mL
VWF:Rco/VWF antigen ratio	0.53	/	/	>0.7

## Data Availability

This article, being a case report and review, does not contain any primary data for sharing. The data discussed are derived from previously published studies and the patient’s medical records.
